# Particular swelling on the palate

**DOI:** 10.11604/pamj.2018.30.280.16529

**Published:** 2018-08-20

**Authors:** Cinzia Casu, Riccardo Botta

**Affiliations:** 1 DDS, Private Dental Practice, Cagliari, Italy; 2 Departement of Dentistry, IRCSS San Raffaele Hospital, Dental School, University of Milano, Milano, Italy

**Keywords:** Oral vascular lesion, palatal swelling, palatal lesions

## Image in medicine

A 75-year-old patient came to our observation for a follow-up visit. We note the presence on the soft palate of a big swelling and the patient reported the presence of this lesion for about 10 years. At the dental level it was possible to see some residual dental elements and the result of recent dental extractions. The anamnesis reported a slight hypertension, osteoporosis and difficulty in swallowing probably linked to the presence of swelling, which however was asymptomatic. It was decided to perform an ultrasound exam to discover the nature of the injury. The ultrasound showed an echogenic area of 37 x 20mm in diameter, with a well-defined oval structure and borders, widely vascularize. The report was compatible with vascular injury. The patient underwent surgery at a hospital, where the lesion was aspirated. We do not have the needle aspiration report and we have not seen the patient after. The differential diagnosis is with an infection and inflammation, oral lymphoma, salivary glands tumors.

**Figure 1:**
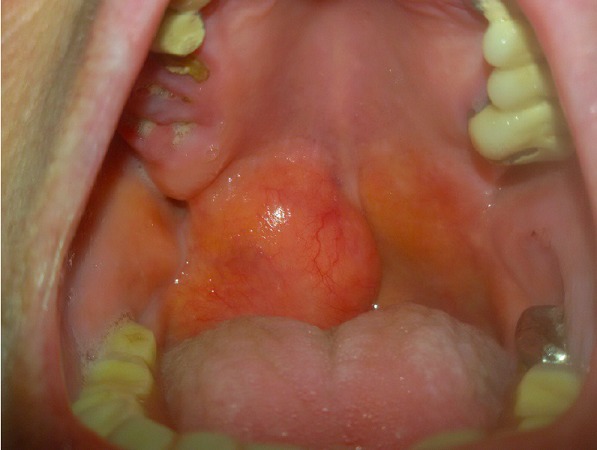
palatal vascular lesion

